# Orbital Rhabdomyosarcoma in a Young Adult With Prior Childhood Leukaemia: A Case Report

**DOI:** 10.7759/cureus.102528

**Published:** 2026-01-28

**Authors:** Ling Paulina Gronczewska, Osman Haji, Raza A Syed, Ibrahim Basar

**Affiliations:** 1 Department of General Medicine, West Suffolk Hospital, Bury St Edmunds, GBR; 2 Department of General Medicine, National Health Service (England), London, GBR; 3 Department of Ophthalmology, West Suffolk Hospital, Bury St Edmunds, GBR; 4 Department of General Medicine, Princess Alexandra Hospital, Harlow, GBR

**Keywords:** leukaemia, orbital mass, orbital rhabdomyosarcoma, orbital rms, rhabdomyoscarcoma

## Abstract

Rhabdomyosarcoma (RMS) is a rare and aggressive soft tissue malignancy primarily affecting children and adolescents. Orbital involvement is an uncommon presentation, particularly in young adults. In this case, we present a 21-year-old male with a history of childhood leukaemia treated with chemotherapy and bone marrow transplantation, who presented with a rapidly growing medial orbital mass subsequently diagnosed as RMS. The case emphasises the difficulties in diagnosing the condition, the value of early imaging and biopsy, and the complexities in managing orbital RMS in a patient with a significant oncological history.

## Introduction

Rhabdomyosarcoma (RMS) is an aggressive malignant soft-tissue tumour arising from skeletal muscle progenitors and represents the most common soft-tissue sarcoma of the head and neck in childhood, with approximately 10% of cases involving the orbit [1,2]. Although predominantly a paediatric malignancy, RMS is rare in adults and is associated with poorer outcomes compared with childhood disease [3]. Because of its aggressive nature and propensity to cause rapid visual and structural deterioration, early recognition and prompt multidisciplinary management are essential.

RMS comprises several histological subtypes, most commonly embryonal and alveolar variants, with embryonal tumours predominating in the head and neck region. Orbital disease typically presents with rapidly progressive proptosis, eyelid or medial canthal swelling, diplopia, raised intraocular pressure, and potential optic nerve compromise - features that can closely mimic more common infectious or inflammatory orbital disorders encountered in general practice [4,5]. These overlapping presentations may delay diagnosis unless clinicians maintain a high index of suspicion.

Survivors of childhood leukaemia treated with intensive chemotherapy, radiotherapy, or stem-cell transplantation are recognised to be at increased risk of therapy-related secondary malignancies due to treatment-induced mutagenic effects on developing tissues. Such secondary cancers may include haematological malignancies and solid tumours, including sarcomas, emerging years after the initial therapy.

We report a rare case of adult-onset orbital RMS in a young adult with a history of childhood leukaemia treated with chemotherapy and bone marrow transplantation, highlighting its atypical presentation, potential therapy-related aetiology, and the diagnostic and management challenges for non-specialist clinicians.

## Case presentation

History

A 21-year-old male presented to the ophthalmology clinic at a District General Hospital on February 17, 2025, with a two-week history of swelling in the left medial canthal area, associated with intermittent fever and recent coryzal symptoms. He reported progressive diplopia, particularly on lateral gaze. There was no history of trauma or visual loss.

His past medical history was significant for acute leukaemia diagnosed at the age of four, for which he was treated with chemotherapy and underwent two bone marrow transplants. He also had a history of bilateral avascular necrosis of the hips, requiring hip replacements.

Examination

**Figure 1:**
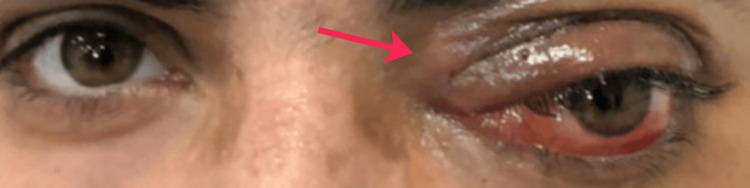
Clinical photograph demonstrating a firm medial canthal mass (arrow), associated left-sided proptosis, and inferolateral displacement of the globe

On inspection and palpation, there was a tender, firm mass in the left medial canthal region associated with proptosis and inferolateral displacement of the globe (Figure 1). Extraocular movements were restricted in all directions in the left eye.

Best-corrected visual acuity was 6/4.8 in the right eye and 6/24-1 in the left. Colour vision testing revealed 15/15 in the right eye and 9/15 in the left. Intraocular pressure measured 18 mmHg in the right eye and 38 mmHg in the left. Marked chemosis and punctate epithelial changes were present in the left eye. No relative afferent pupillary defect was detected (Table 1).

**Table 1:** Clinical summary and diagnostic findings for a 21-year-old male with orbital rhabdomyosarcoma with reference ranges and normal values

Parameter	Result	Reference Range/Normal Values	Comment
Best Corrected Visual Acuity	Right eye: 6/4.8, Left eye: 6/24-1	6/6 (20/20) for each eye	Decreased visual acuity in the left eye
Colour Vision Testing	Right eye: 15/15, Left eye: 9/15	15/15 for each eye	Decreased colour vision in the left eye
Intraocular Pressure (IOP)	Right eye: 18 mmHg, Left eye: 38 mmHg	10-21 mmHg for each eye	Elevated IOP in the left eye

Investigations

Given the acute presentation with concern for infectious or inflammatory pathology, and the need to rapidly assess bony involvement of the medial orbital wall and paranasal sinuses, a contrast-enhanced CT was selected as the first-line imaging modality. The contrast-enhanced CT scan of the orbit was performed on February 18, 2025, which demonstrated a soft tissue mass originating from the ethmoid sinus with erosion through the medial orbital wall. The lesion extended into the orbit, exerting mass effect on the globe and extending posteriorly towards the orbital apex.

Although the patient initially reported coryzal symptoms and intermittent fever, several features were considered atypical for uncomplicated inflammatory orbital disease, including the rapid progression of a firm medial canthal mass, early proptosis, marked extraocular movement restriction, elevated intraocular pressure, and deterioration in visual function. Initial differential diagnoses included orbital cellulitis, paranasal sinus mucocele, lymphoma, and other malignant orbital tumours, prompting urgent cross-sectional imaging and biopsy.

Management

The patient was started on oral co-amoxiclav and listed for urgent orbital biopsy, performed on February 19, 2025, under local anaesthetic. A pre-septal approach was used to access the lesion within the medial orbit. The sample was sent for histological analysis. Immediate postoperative recovery was uneventful.

Histopathological findings revealed a “small round blue cell” tumour morphology. Immunohistochemical staining was consistent with RMS. The diagnosis was subsequently confirmed by the pathology team at Addenbrooke’s Hospital.

Following confirmation, the patient was referred urgently to the Oncology and Oculoplastics teams at Addenbrooke’s Hospital for staging and further management. At follow-up, sutures were removed, and the patient continued to experience diplopia and orbital discomfort.

Follow-Up

On review at Cambridge University Hospitals on March 4, 2025, there was a significant worsening of proptosis, chemosis, and orbital discomfort. Intraocular pressure in the left eye was 31 mmHg, and ocular motility remained markedly limited, with further reduction in visual acuity. This acute deterioration raised concern for either post-biopsy inflammatory oedema or rapid tumour progression, both of which carried a risk of optic nerve compression and permanent visual loss. These visual prognosis concerns prompted urgent multidisciplinary discussion with the oncology and oculoplastics teams, and interim management included intensive ocular lubrication and close monitoring.

Diagnosis

A multidisciplinary review was subsequently held at University College London Hospital (UCLH) on April 11, 2025, to discuss the case. The consensus confirmed the diagnosis of orbital RMS and recognised the potential for a therapy-related secondary malignancy given the patient’s oncological history. The team agreed on initiating proton beam radiotherapy as part of definitive management, aiming to deliver local tumour control while minimising radiation exposure to surrounding ocular and neural structures. A course of proton beam therapy was planned and scheduled at a tertiary radiotherapy centre, with close coordination between oncology, radiology, and ophthalmology services for ongoing review.

## Discussion

Orbital RMS is uncommon in adults and usually manifests in children [1-3]. The diagnostic challenge in this case lies in the subtle and nonspecific early presentation. Upper eyelid swelling is a common finding in ophthalmic practice and is often attributed to benign causes such as infection or inflammation [4]. However, it was crucial in this case to identify red flag features, including the presence of minimal early proptosis and a palpable firm mass in the medial canthal region [5]. These subtle but significant findings prompted further investigation and were key to reaching the diagnosis.

This case underscores the need for clinicians to maintain a high index of suspicion for orbital RMS when evaluating young adults with rapidly progressive periocular swelling that may initially mimic infection, particularly in those with a history of childhood malignancy or bone marrow transplantation. The patient’s oncological background raised concern for a therapy-related secondary malignancy and lowered the threshold for aggressive investigation [3]. The tumour’s aggressiveness was reflected by the rapid progression of symptoms, including increasing proptosis and ocular discomfort over a short period.

The early stages may be deceptive, with preserved visual acuity and absence of an afferent pupillary defect masking the underlying severity. The ethmoidal origin with invasion into the orbit and paranasal sinuses reflects the embryonal subtype’s infiltrative behaviour. Biopsy remains essential for diagnosis, with histology demonstrating the characteristic small round blue cell morphology.

A prompt multidisciplinary approach involving ophthalmology, oncology, pathology, and radiotherapy teams was vital to guide management. Referral to a tertiary oncology centre allowed for appropriate staging and treatment planning, including proton beam radiotherapy, to achieve local control while minimising damage to surrounding ocular and neural structures.

Population-based analyses have demonstrated a lower incidence of RMS in adults compared with paediatric patients, and orbital involvement outside childhood is uncommon, with most descriptions arising from small orbital series and reviews [1-3]. Compared with these reports, our patient was notable for adult age, prior childhood malignancy treated with bone marrow transplantation, ethmoidal sinus origin with orbital invasion, and rapid progression mimicking inflammatory disease, ultimately requiring proton beam radiotherapy.

## Conclusions

This case is notable for the occurrence of orbital RMS in a young adult with a history of childhood leukaemia treated with chemotherapy and bone marrow transplantation, raising concern for a therapy-related secondary malignancy. Orbital RMS in young adults is rare but should be considered in rapidly progressing orbital masses, particularly in patients with a history of childhood malignancy. While this report describes a single patient and therefore cannot support broad prognostic or therapeutic generalisations, it reinforces important clinical warning signs and diagnostic principles relevant to general practice. Early imaging, biopsy, and multidisciplinary referral are key to timely diagnosis and treatment. This case also highlights the importance of recognising post-biopsy symptom progression and the need for close postoperative monitoring.
